# Differential HIF-1α and HIF-2α Expression in Mammary Epithelial Cells during Fat Pad Invasion, Lactation, and Involution

**DOI:** 10.1371/journal.pone.0125771

**Published:** 2015-05-08

**Authors:** Sven Påhlman, Leif R. Lund, Annika Jögi

**Affiliations:** 1 Department of Laboratory Medicine Lund, Translational Cancer Research, Lund University Cancer Center at Medicon Village, Lund University, Lund, Sweden; 2 Department of Cellular and Molecular Medicine, Faculty of Health Sciences, University of Copenhagen, Copenhagen, Denmark; Institute of Biomedicine, FINLAND

## Abstract

The development and functional cycle of the mammary gland involves a number of processes that are caricatured by breast cancer cells during invasion and metastasis. Expression of the hypoxia-inducible transcription factors HIF-1 and HIF-2 has been associated with metastatic, poor prognosis, and high-grade breast cancers. Since hypoxia affects normal epithelial differentiation, we hypothesise that HIFs are important for normal breast epithelial development and regeneration as well as cancer initiation and progression. Here, we investigated the expression of the oxygen-sensitive HIF-alpha subunits during mouse mammary gland development, lactation, and involution. In breast epithelial cells, HIF-1α was expressed during early development, prior to cell polarisation. In contrast, expression of HIF-2α occurred later and was restricted to a subpopulation of luminal epithelial cells in the lactating gland. Mammary gland involution is a developmental stage that involves extensive tissue remodelling with cell death but survival of tissue stem/progenitor cells. At this stage, HIF-2α, but little HIF-1α, was expressed in CK14-positive epithelial cells. The temporal but differential expression of the HIF-alpha subunits during the mammary gland life cycle indicates that their expression is controlled by additional factors to hypoxia. Further functional studies of the roles of these proteins in the mammary gland and breast cancer are warranted.

## Introduction

In contrast to the development of most organs, mammary gland development not only takes place during embryogenesis and foetal life, but also during three distinct phases: embryonal development, puberty, and pregnancy [1]. The mammary epithelial anlage forms during embryogenesis and resides just beneath the nipple. During puberty, the ductal tree forms by extensive epithelial proliferation and invasion of the mammary fat pad. Further branching of the ductal tree takes place during early pregnancy, when milk-producing alveoli are formed and, towards the end of pregnancy, final differentiation takes place and milk production is hormonally induced. During lactation, the functional part of the mammary gland lifecycle, intensive metabolism is stimulated by hormones and suckling. After weaning, the mammary gland is remodelled into a state similar to the virgin gland via a process termed involution. Importantly, mammary epithelial stem and progenitor cells with the capacity to re-build the functional mammary gland in subsequent pregnancies survive this process, which involves extensive cell death. Similar, but less fulminant, cycles of growth and involution take place during the monthly oestrous cycle in women [1,2]. These processes of mammary gland development and function are similar in humans and mice [3].

Breast cancer represents a caricature of the features seen in the developing and functional mammary epithelium. The malignant hallmark of invasive growth is similar to the epithelial invasion of the mammary fat pad seen in puberty and early pregnancy. Metabolism is changed during lactation and during malignant growth. Epithelial confinement to the basement membrane is lost in cancer, while during normal epithelial invasion of the mammary fat pad the basement membrane has yet to be established and in involution it is loosened by proteolysis. A population of epithelial cells with stem cell traits that can grow and overcome anoikis exists in cancers, processes that are also seen in mammary gland involution. Despite the protective effects of pregnancy and breast-feeding on lifetime cancer risk, a temporal increase in breast cancer risk occurs during pregnancy and childbirth. Furthermore, pregnancy-associated breast cancer has a worse prognosis [4].

The hypoxia-inducible transcription factor subunits HIF-1α and HIF-2α are associated with breast cancer metastasis and poor patient survival [5,6]. We recently showed that hypoxia and HIF transcriptional activity are linked to a state of loss of polarisation and a cancer-like phenotype in primary human breast epithelial cells [7,8]. Selective silencing of HIF-1α expression in mouse mammary epithelium and mouse breast cancer models [9–11] led to failed lactation and increased tumour growth and metastasis, respectively. HIF-2α expression has yet to be selectively targeted in the mammary epithelium. Both HIF-1α and HIF-2α are primarily regulated post-translationally, with immediate proteosomal protein degradation via ubiquitination by the ubiquitin ligase pVHL in the presence of oxygen [12]. In addition, the expression of HIF-α subunits can be regulated by growth factors and oncogenes [13,14]. Silencing of pVHL expression in mammary epithelium leads to stabilisation of both HIF-1α and HIF-2α accompanied by hyper-proliferation and an inability to support lactation in serial pregnancies, consistent with depletion of the stem cell pool [15]. Interestingly, concurrent targeting of HIF-1α expression does not rescue the pVHL-null phenotype, which might suggest a role of HIF-2α in the maintenance of the stem cell pool.

Here, we analyse the protein expression of HIF-1α and HIF-2α in mammary gland development, function, and involution. Our data uncover specific expression of HIF-1α and HIF-2α in distinct subsets of mammary epithelial cells at precise stages during organ development and function. The divergent expression patterns of the two factors suggest that their regulation is not merely due to oxygen availability.

## Material and Methods

### Ethics Statement

All mouse works were ethically approved by national and institutional guidelines. Animal care at the University of Copenhagen and Copenhagen University Hospital, Copenhagen, Denmark was in accordance with national and institutional University of Copenhagen and Copenhagen University Hospital, Copenhagen, guidelines. The current study was approved by the Danish Animal Experiments Inspectorate (permissions#2007/561-1053). Mice were anaesthetised by intra peritoneal injection of 0.03 ml/10 g of a 1:1 mixture of Dormicum (Midazolam 5 mg/ml) and Hypnorm (Fluanison 5 mg/ml and Fentanyl 0.1 mg/ml). All mice were free from murine pathogens in accordance with the FELASA recommendations for health monitoring of experimental units [16].

### Animals and tissue treatment

To isolate mammary glands, mice were anaesthetised by intraperitoneal (i.p.) injection of 0.03 ml/10 g of a 1:1 mixture of Dormicum (midazolam 5 mg/ml) and Hypnorm (fluanison 5 mg/ml and fentanyl 0.1 mg/ml). Mice were intracardially perfused with 10 ml of ice-cold phosphate-buffered saline (PBS) and 10 ml 4% (w/v) paraformaldehyde (PFA). The fourth abdominal mammary glands were then recovered and fixed for 16 h in 4% PFA. Tissue was stored in 70% ethanol for up to 24h, rinsed in PBS, dehydrated, and embedded in paraffin (Histokinette).

Animal care at the University of Copenhagen and Copenhagen University Hospital, Copenhagen, Denmark was in accordance with national and institutional guidelines (permissions #2007/561-1053), and all mice were free from murine pathogens in accordance with the FELASA recommendations for health monitoring of experimental units [16].

The mouse mammary gland material consisted of the fourth gland from 45 C57BL/6J mice: 5 aged 30 days, 6 aged 35 days, 6 aged 50 days, 6 aged 70 days, 4 lactating day 7, 5 lactating 7 days and 1 day involution, 3 lactating 7 days and 2 days involution, 4 lactating 7 days and 5 days involution, 3 lactating 7 days and 7 days involution, and 3 lactating 7 days and 14 days involution. Litter sizes were harmonised and, after 7 days of lactation, forced involution was induced by removal of the pups.

Hypoxyprobe-1 (pimonidazole salt, Hypoxyprobe, USA) 1.5 mg/mouse (25g body weight) was injected i.p. in PBS one hour prior to mouse sacrifice and intracardial perfusion. This was performed in MMTV-PymT heterozygous 12 weeks old female C57BL/6J mice with tumours.

### Cell culture

Breast cancer cells, MCF-7 (ATCC), were grown in standard DMEM/F12 (1:1) medium (Thermo Scientific) with FCS (1%, Biosera), penicillin & streptomycin (100 units/ml, Hyclone) and insulin (100 units/ml, Actrapid). The cells were routinely cultured at 37°C, 5% CO_2_ and air oxygen levels. Experimental hypoxic cell culture was performed for 72h in a DonWhitney Hypoxystation under 1% oxygen concentration and otherwise same culture conditions as described above. The adherent cells were rinsed with ice-cold PBS, covered with 4% PFA in PBS and then scraped off the culture dish with a cell scraper. After 15 min the cells were stained for 1 min with Mayer’s hematoxylin, gently centrifuged and fixation was removed and the cells dehydrated and embedded in paraffin. Sections were subjected to IHC employing the same conditions as the tissue sections, see below.

### Immunohistochemistry (IHC)

Tissue sections were deparaffinised in xylene and hydrated through graded ethanol/water dilutions. Antigen retrieval was performed by microwaving at 98°C for 15 min in citrate buffer (10 mM, pH 6); this treatment also prevents recognition of endogenous IgG by anti-mouse antibodies. Endogenous peroxidase activity was blocked using 1% hydrogen peroxide for 15 min at ambient temperature. Sections were then washed in running tap water for 3–5 min and in Tris-buffered saline (TBS: 50 mM Tris, 150 mM NaCl, pH 7.6) containing 0.5% Triton X-100 (TBS-T) for 5 min. Slides were mounted into racks with immunostaining coverplates (Dako Cytomation, Denmark) for subsequent incubations. Slides were incubated 1h with primary antibody. The secondary antibody was EnVision+ anti-mouse/rabbit. For rat F4/80 monoclonal antibody, a middle step of rabbit anti-rat IgG (Dako) was employed. The antibodies used were (company, dilution): anti-collagen IV (Abcam, Ab6586, 1:500), anti-HIF-1α (Novus Biologicals 493, 1:1000), anti-HIF-2α (Novus Biologicals 100–132, 1:250), CK8 (AbCam, 1:200), CK14 (Covance Research 155p, 1:250), F4/80 (Abcam, 1:250), Hypoxyprobe antisera (Hypoxyprobe, 1:1500). Negative controls were prepared in parallel without primary antibody.

For double IHC staining, the same antibodies as described above for HIF-2α, CK8, CK14, and F4/80 were used with Polink double IHC staining kits (BIOSS) according to the manufacturer’s instructions.

#### Microscopy

Microscopy was performed using an Olympus (Olympus) and a Nikon Fusion microscope (Nikon) with 10x, 20x, and 40x objective lenses as stated in the figure legends. Image acquisition was performed with Cell^A^ imaging software and there was no non-linear image manipulation.

## Results

### Evaluation of HIF antibodies

**Fig 1:**
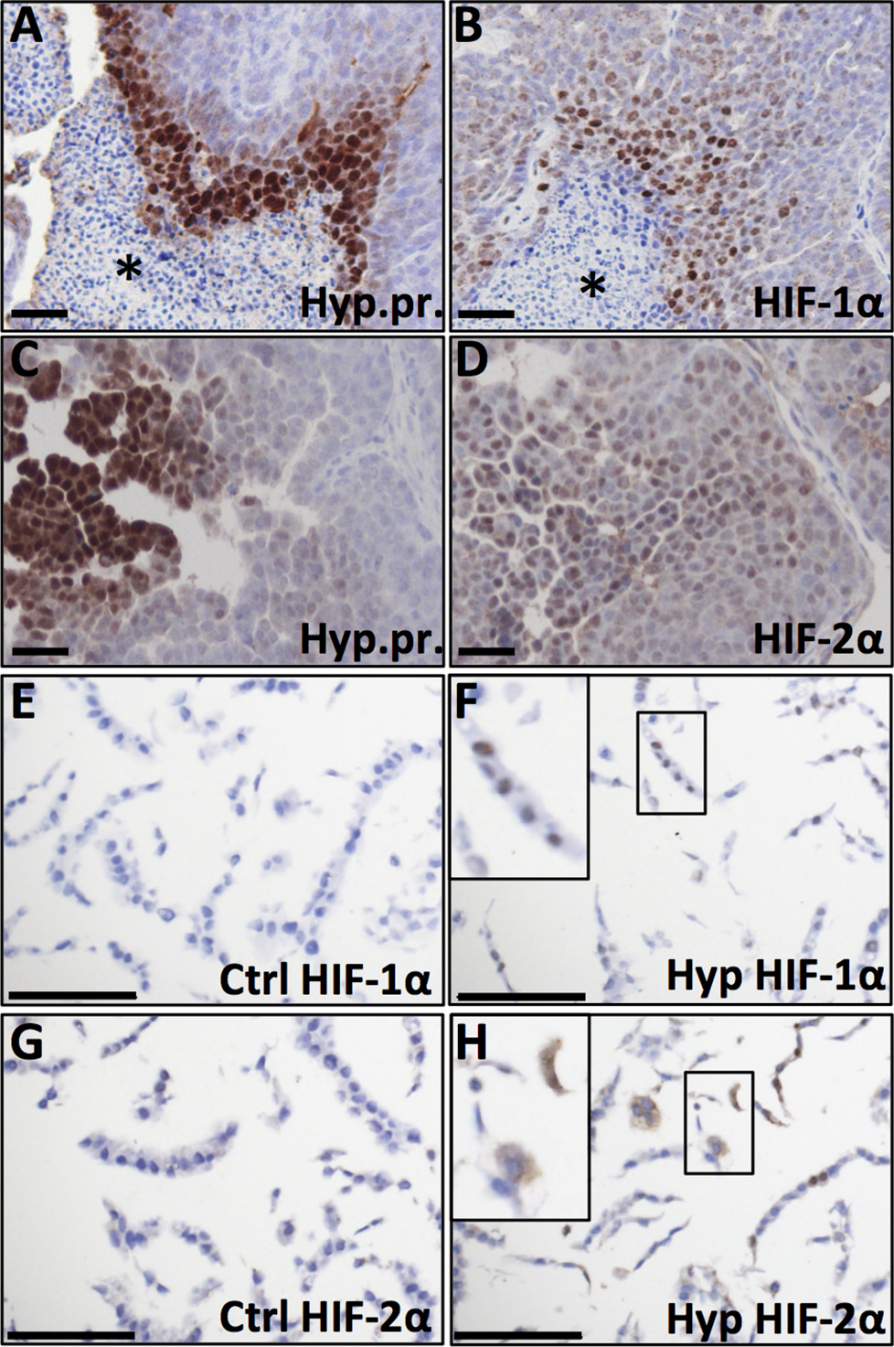
HIF-1α and HIF-2α IHC signals in hypoxic areas of transgenic mouse mammary tumours. **A, C** Hypoxyprobe (Hyp.pr.) allows visualisation of hypoxic tumour areas. **B**. Hypoxic peri-necrotic HIF-1α-positive cells display nuclear staining. **D**. HIF-2α-positive cells show cytoplasmic staining with or without appreciable nuclear staining. * necrosis. **E, F**. IHC staining for HIF-1α on MCF-7 breast cancer cells grown under control (E) and hypoxic (F) conditions respectively. **G, H**. Control (G) and hypoxic (H) MCF-7 cells IHC stained for HIF-2α. 20x obj. Insets show magnification of the boxed area. Size bars 50 μm.

Immunohistochemical detection of HIF-alpha subunits, especially HIF-2α, can be challenging. We therefore investigated the HIF staining pattern of the antibodies used in this study using tumours obtained from a transgenic mouse mammary tumour model (MMTV-PyMT) injected with Hypoxyprobe (Hypoxyprobe, USA) as reference tissue. Both HIF-1α and HIF-2α proteins were selectively detected in hypoxic peri-necrotic areas (Fig 1). HIF-1α showed nuclear localisation (Fig 1B), consistent with binding to DNA under hypoxic conditions [12,17]. The HIF-2α signal was both nuclear and cytoplasmic in hypoxic regions, but cytoplasmic staining was not always accompanied by appreciable nuclear signal (Fig 1D). In a second approach to verify the IHC with the HIF-1α and HIF-2α antibodies we cultured MCF-7 breast cancer cells under control (21% oxygen) and hypoxic (1% oxygen) conditions for 72 h. These cells showed no expression of HIF-1α under oxygenated conditions (Fig 1E) and nuclear HIF-1α accumulation in a large proportion of cells at hypoxia (Fig 1F). Occasional MCF-7 cells were positive for HIF-2α under oxygenated conditions (Fig 1G) and with hypoxia the number of HIF-2α positive cells increased much (Fig 1H) in line with our previous observations [6]. HIF-2α signal was seen both in the nucleus and in the cytoplasm and some cells had expression in both compartments whereas others had expression in the cytoplasm without appreciable nuclear signal (Fig 1). The cytoplasmic HIF-2α signal was consistent with previous findings in neuroblastoma [18] and the recently described role for HIF-2α in the hypoxic regulation of translation by binding to mRNA in the cytoplasm [19]. We conclude that the antibodies and conditions used specifically detect HIF-1α and HIF-2α, respectively.

### HIF-alpha subunit expression in virgin mouse mammary glands

At puberty, hormonal stimulation induces the mammary epithelium to proliferate and form ductal structures that invade the mammary fat pad. The tip of the growing duct invades the mammary fat pad and, at this stage in development, there is no basement membrane to separate the epithelial and mesenchymal compartments, similar to invasive cancer. Invasion of the epithelial cells into the fat pad requires the timely expression and activation of extracellular proteolytic factors such as matrix metalloproteases (MMPs) and urokinase plasminogen activator (uPA) and its cell surface receptor uPAR [1,20,21]. MMPs, uPA, and uPAR can be transcriptionally induced by HIF-1α and HIF-2α. The invasive growth of the mammary epithelium into pre-existing tissue shares numerous features with the invasive growth and dissemination of mammary cancer, and, in this malignant setting, the activation of HIF-induced transcription is implicated in extracellular proteolytic activity, invasion, and angiogenesis [12,22].

**Fig 2:**
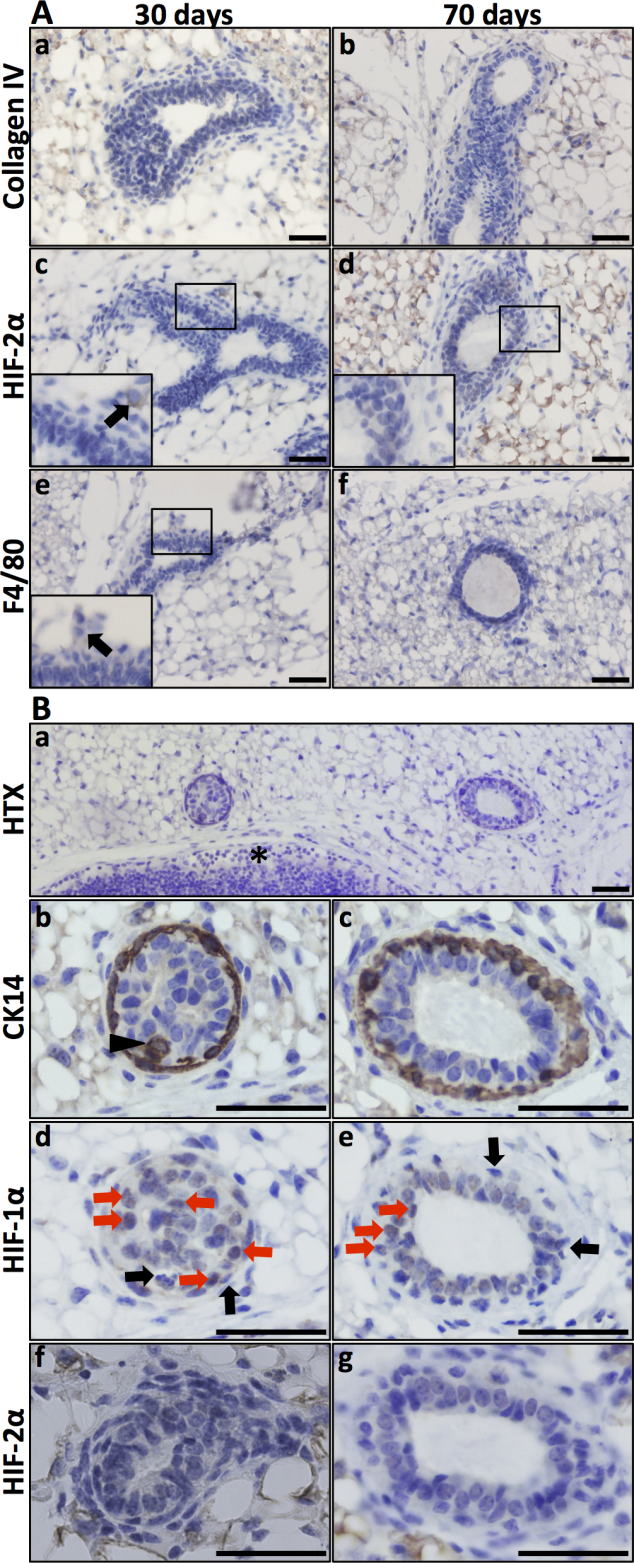
HIF-1α and HIF-2α expression in the virgin mammary gland. **A**. Virgin mammary glands (30 and 70 days old) showed no conspicuous basal membrane, as visualised by collagen IV IHC (**a, b**). There was also no detectable expression of HIF-2α in the epithelial cells (**c, d**). Macrophages (F4/80 positive) were few. In panel c, a single HIF-2α-positive cell was detected, and the adjacent F4/80 IHC section (**e**) suggested that this cell is a macrophage. **B**. Expression of HIF-1α in mammary epithelium in the 70-day-old virgin mouse. Top panel, orientation slide with haematoxylin (HTX) staining, 20x obj. *lymph node. **Panels b, d, f**. Cross-section of a developing duct close to the invading tip at a stage where the lumen is not yet evacuated, 40x obj. **Panels c, e, g**. Cross-section of a less mature part of a duct, 40x obj. CK14-expressing cells (marker of basal mammary epithelial cells) can be seen in more than one cell layer (panels b and c, arrow-head). At this stage, the lumen is evacuated, but there is still more than one layer of epithelial cells. HIF-1α IHC on the adjacent sections (panels d, e) showing nuclear expression in non-basal epithelial cells (highlighted by red arrows). Basal (CK14 positive) epithelial cells did not express HIF-1α (black arrows). Mammary epithelial expression of HIF-2α was not detected at these developmental stages. Size bars: 50 μm.

**Fig 3:**
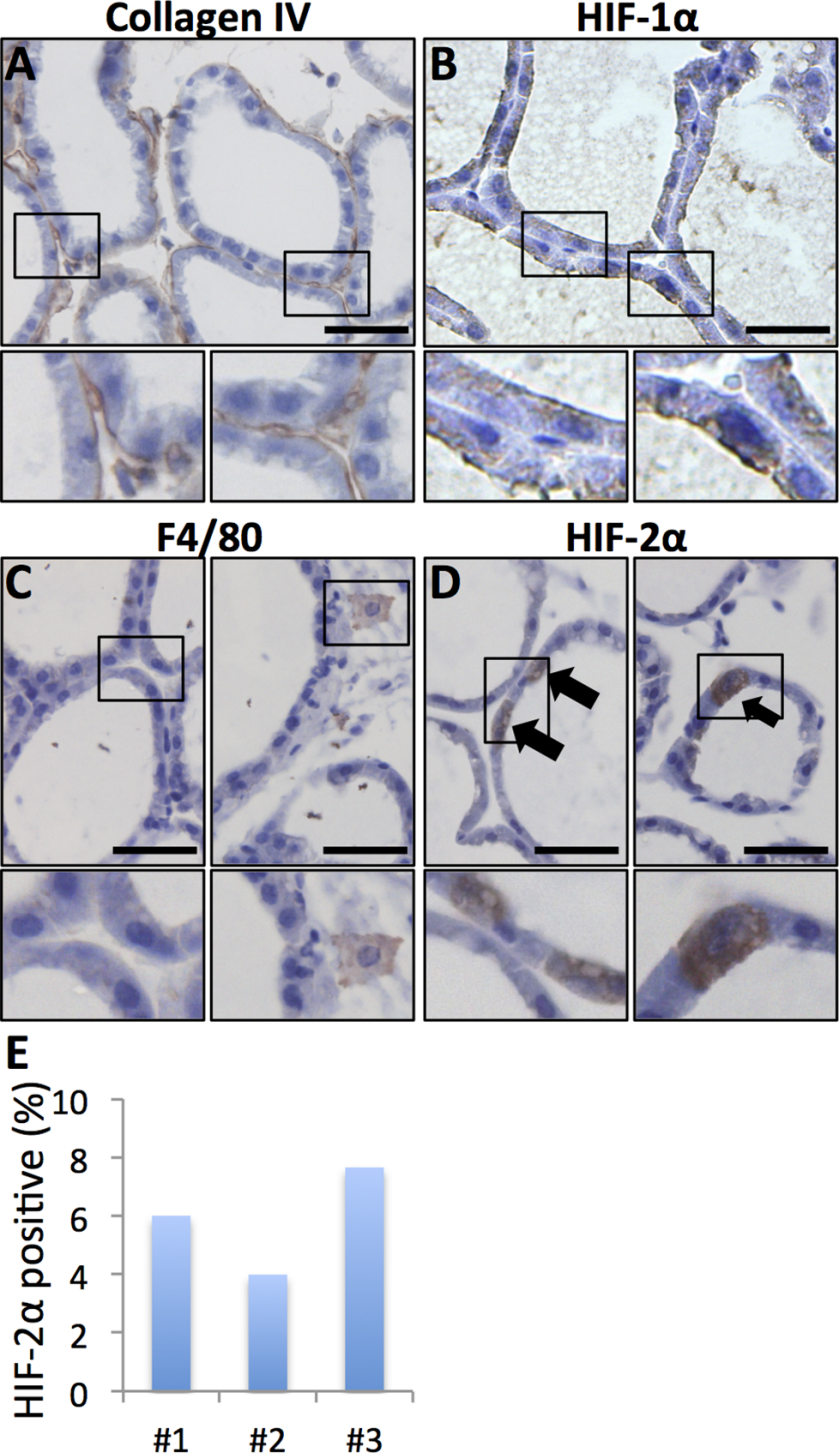
Lactating mammary gland. Smaller panels display enlargements of the indicated areas. Size bars: 50 μm, 40x obj was used for all micrographs. **A**. Collagen I IHC allows visualisation of the basement membrane surrounding the dilated ducts. **B**. HIF-1α was not detected in the epithelial cells of the lactating gland (compare with Fig 1). **C**. Macrophage infiltration was sparse in the lactating mammary gland as judged by F4/80 IHC. **D**. A subset of cuboidal luminal epithelial cells was distinctively positive for HIF-2α. **E**. The percentage of HIF-2α-positive out of total luminal epithelial cells was counted in sections from three mice.

The expression and function of HIF-2α in the developing mammary epithelium is less well characterised compared to that of HIF-1α [9,10]. To investigate the expression of HIF-2α in the mouse mammary gland during development, lactation, and involution, we first stained tissues collected at these stages for collagen IV expression to visualise the basement membrane and general duct structure (Figs 2 and 3). Several layers of epithelial cells surrounded the forming lumen, and the collagen-containing basement membrane was not yet visible in the early virgin mammary gland. In these structures, HIF-2α was not detected in the mammary epithelial cells (Fig 2A and 2B). In the displayed micrograph of pubertal mammary ducts (Fig 2Ac) there was one HIF-2α positive cell and an adjacent section IHC stained for F4/80 (Fig 2Ae) suggests that this cell was a macrophage (often HIF-2α positive). During puberty macrophages infiltrate the mammary fat-pad and localise to the area close to the ductal terminal end buds [23]. Since several proteins (e.g., MMPs and uPAR) are known to be HIF induced and are expressed during epithelial invasion of the mammary fat pad [20], we stained these early glands for HIF-1α. In line with these observations, HIF-1α-positive cells were detected in the epithelial cells of virgin mammary ducts (Fig 2B).

To determine which epithelial cells expressed HIF-1α at these early time points, we next stained for the basal epithelial marker CK14. As exemplified by the day 70 mammary glands, CK14 was expressed in the basal epithelium (Fig 2) of epithelial structures where the lumen is not yet evacuated (Fig 2Bb) and where the lumen has been evacuated (Fig 2Bc). In contrast, HIF-1α was expressed in the luminal epithelial cells, as defined by microscopic localisation and lack of CK14 immunoreactivity (Fig 2B). In these structures, HIF-1α and CK14 expression was mutually exclusive (Fig 2B; black arrows indicating examples of cells positive for CK14 but lacking HIF-1α expression). HIF-2α was undetectable in mammary epithelial cells in additional virgin glands (Fig 2B).

### HIF-2α is expressed in a subset of luminal epithelial cells in the lactating mammary gland

Epithelial cells undergo full differentiation during lactation, at which time metabolism is extremely high as energy-rich milk is produced. The lactating gland mainly consists of luminal epithelial cells and open dilated lumina, the basal cell compartment is sparse compared to luminal cells, and the fat cells are depleted and hardly detectable (9, 12, Fig 3).

Although HIF-2α was not expressed at detectable levels in virgin mouse breast epithelium, expression was apparent during lactation. Distinct, mostly cytoplasmic expression of HIF-2α was detected in a subpopulation of luminal epithelial cells (Fig 3). These HIF-2α-positive cells were cuboidal, similar to adjacent non-HIF-2α-expressing luminal epithelial cells (Fig 3). The fractions of luminal epithelial cells positive for HIF-2α were calculated in the fourth mammary glands from three different mice, three randomly placed fields per gland, and ranged between 4 and 8% (number of HIF-2α positive luminal epithelial cells/number of luminal epithelial cells:17/283, 19/559, and 20/261; Fig 3E). Macrophages were very scarce in the lactating glands, but could be found when actively searched for (Fig 3C). Importantly, the HIF-2a-positive cells in the epithelial layer were not macrophages as assessed by their morphological appearance and negativity for the F4/80 macrophage marker (Fig 3C). The lactating mammary gland has high metabolic activity, and it is plausible that local hypoxic conditions occur. We therefore analysed these tissues for HIF-1α expression; however, there was no detectable expression suggesting that the expression of HIF-2α in the distinct subpopulation of luminal epithelial cells is not triggered by hypoxia (Fig 3B).

### HIF-alpha subunit expression in mouse mammary glands during involution

After weaning, the mammary gland involutes to a state similar to the virgin gland [1,24–26]. The mammary gland involution is divided into two phases; the reversible phase that encompass approximately the first 72 hours, succeeded by the irreversible or proteinase dependent phase [27]. During the latter phase macrophage infiltration takes place [27]. Most importantly, regrowth potential to form all parts of the functional gland in subsequent pregnancies is retained, i.e., mammary epithelial stem and progenitor cells must be spared during the extensive cell death that occurs during involution [25]. Infiltrating immune cells and other stromal components are the main contributors to the extensive extracellular proteolysis that occurs during involution [20,26,27]. The collagen-containing basement membrane, which is intact and surrounds the dilated ducts during lactation, is disrupted during involution. The epithelial cells show loss of polarity and no longer form organised epithelium.

**Fig 4:**
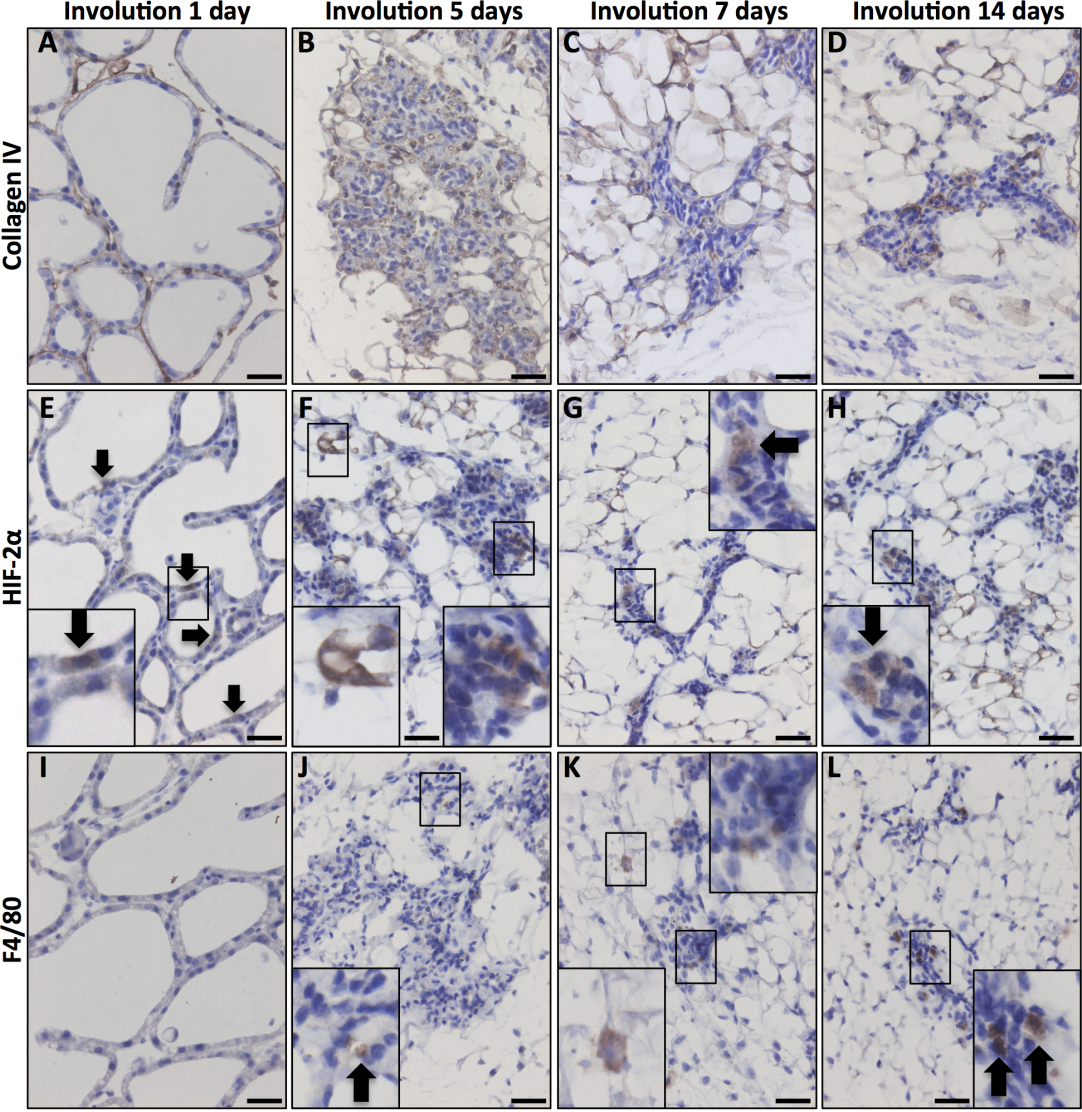
HIF-2α expression in the involuting mammary gland. Inserts are enlargements of the indicated areas. Size bars: 50 μm, 40x obj was used in all micrographs. **A**. In the early involuting gland, the morphology resembles the lactating gland and the basement membrane is evident at this stage. **B-D**. As tissue remodelling proceeds during involution, the collagen layer becomes unstructured. **E-H**. HIF-2α-positive cells were detected at all studied stages of involution. **I-L**. Macrophage infiltration (F4/80 positive) was first evident at the fifth day of involution (J) and increased with time (**K, L**).

**Fig 5:**
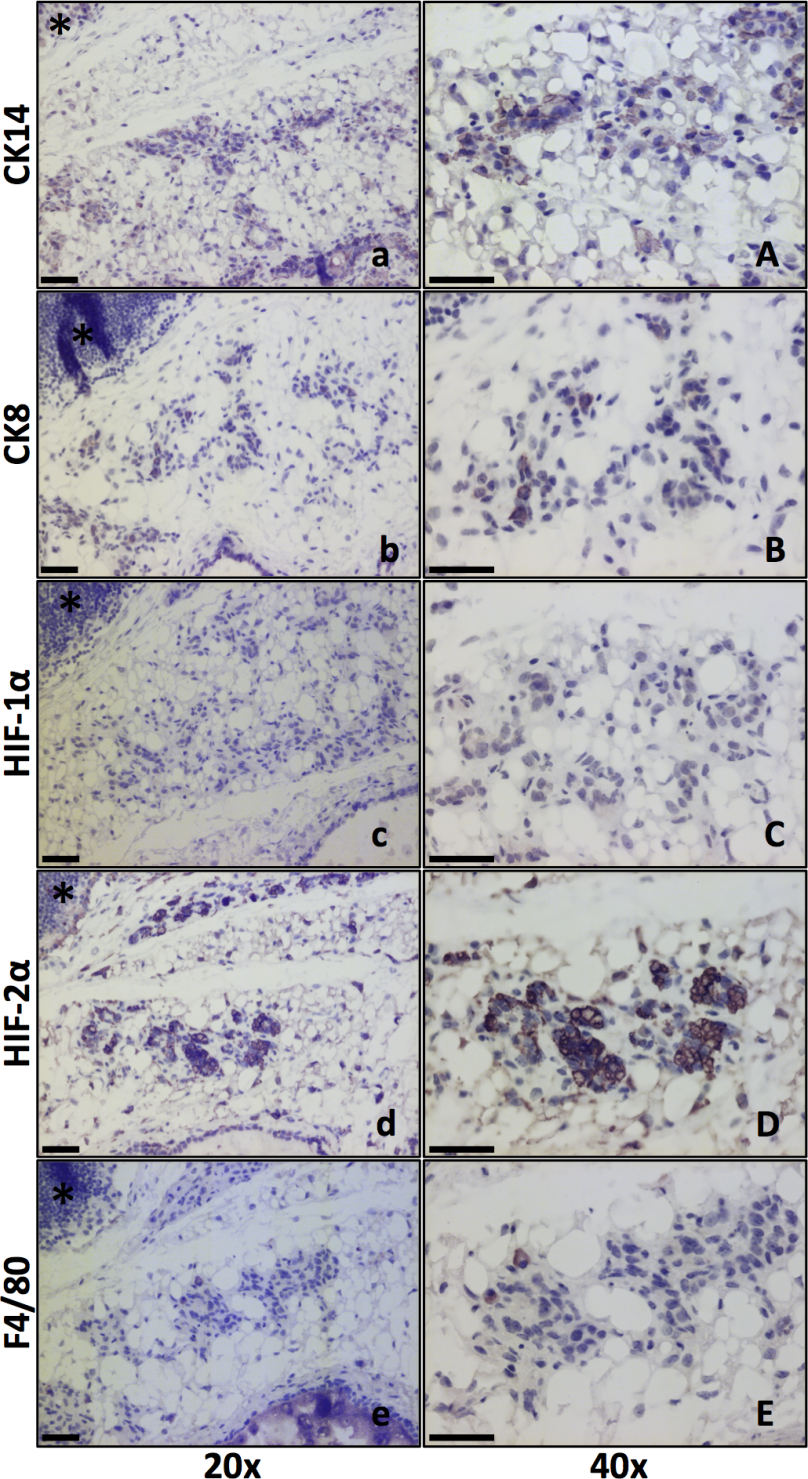
HIF-alpha expression in involuting glands five days post weaning. Size bars: 50 μm. * marks the lymph node for orientation. 20x and 40x lenses as indicated. **a, A**. CK14 marks the basal mammary epithelial cells and stem/progenitor cells. **b, B**. CK8 positivity shows the luminal mammary epithelial cells. **c, C**. HIF-1α IHC showed weak or little positivity in mammary epithelial cells at this stage. **d, D**. HIF-2α positive cells were found in the clusters of epithelial cells. **e, E**. Macrophage (F4/80 positive) infiltration has begun by five days post weaning. F4/80 positive cells were apparently fewer than HIF-2α positive cells.

**Fig 6:**
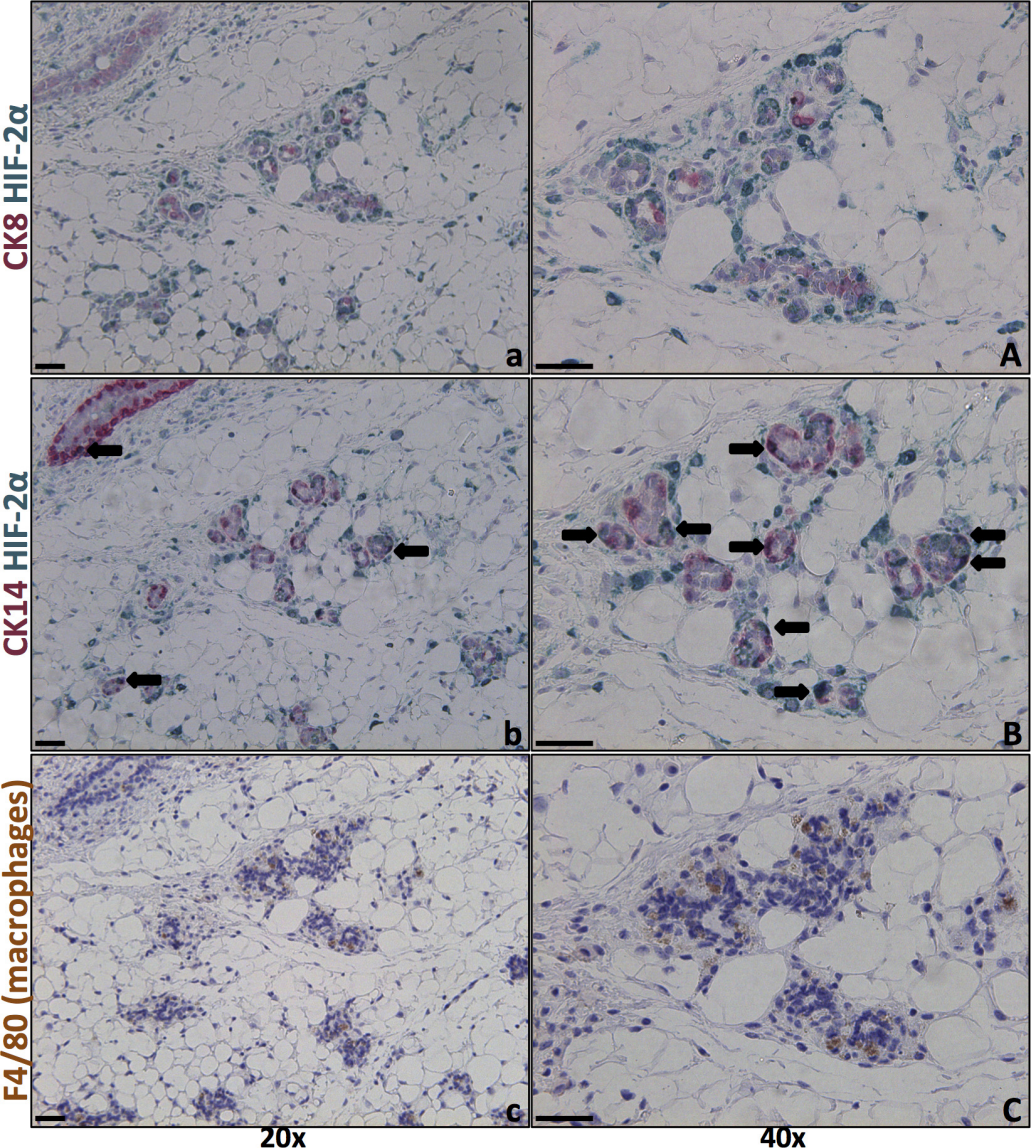
Mainly CK14-positive, but not CK8-positive, mammary epithelial cells are HIF-2α positive at day 14 post weaning. Left panels 20x and right 40x objective. Size bars 50 μm. **a, A, b and B**. Double IHC for HIF-2α (green) and CK8 (a, A) and CK14 (b, B), respectively (red), reveal that few (if any) CK8-positive luminal cells are HIF-2α positive. Numerous CK14-expressing cells, which include basal and stem/progenitor cells, were positive for HIF-2α. **c, C**. F4/80 IHC was performed on an adjacent tissue section detect macrophages.

In the reversible phase of involution one day post weaning, the mammary tissues exhibited similar structure and expression of the studied proteins as during the lactating phase, but shed epithelial cells were apparent in the ductal lumen. In summary, HIF-2α was detected in the cytoplasm in subsets of epithelial cells, while HIF-1α was not detectable and occasional macrophages were seen (Fig 4A, 4E and 4I). As involution and tissue remodelling progressed into the irreversible phase (Day 5 to 14 in Fig 4), the frequency of HIF-2α-positive cells increased (Fig 4F–4H). During these later phases of involution, the ducts collapsed and the basement membrane was degraded and showed scattered collagen IV expression (Fig 4B–4D). Infiltration with F4/80-positive macrophages was also apparent (Fig 4J–4K). The macrophages mainly localised to the edge of the collapsing mammary ducts (Figs 4, 5 and 6), with many of the HIF-2α-expressing cells also localised in the same area (Figs 4, 5 and 6). However, a substantial fraction of the HIF-2α-positive cells were centrally positioned among the CK14- and CK8-expressing mammary epithelial cells (Figs 4, 5 and 6). A small amount of HIF-1α expression could be detected in the mammary gland during late involution (Fig 5C).

CK8 is a marker of luminal epithelial cells, which are the dominant cell type during lactation; therefore, these CK8-positive cells decrease in number during the later phases of involution. In contrast, a higher proportion of the CK14-expressing myoepithelial basal cells remain during involution, with the proportion of these cells increasing [28] (Fig 5). The CK14-expressing mammary epithelial cells are known to include basal epithelial and stem/progenitor cells [29].

To better understand which cell types in the involuting mammary gland expressed HIF-2α, double IHC staining of HIF-2α, CK8, and CK14 was performed, and adjacent sections were stained for the F4/80 macrophage marker. Double IHC for HIF-2α and CK8 showed very few, if any, double-stained cells at five (data not shown) or 14 days of involution (Fig 6). In contrast, a substantial number (but not all) CK14-positive cells were also positive for HIF-2α (Fig 6). Whether these cells are stem/progenitor or basal epithelial cells cannot be determined by CK14 IHC alone.

Immunostaining with the F4/80 macrophage marker suggested that some, but not all, HIF-2α-expressing cells of the involuting gland were macrophages (Fig 6). Interestingly, the majority of detected infiltrating macrophages apparently express HIF-2α (Fig 6).

## Discussion

HIF-2α accumulates and activates in response to hypoxic stress but is also expressed during discrete stages of organ development, for example in the peripheral nervous system [30–32], where it is likely induced by factors other than low oxygen levels. During epithelial invasion of the mammary fat pad, oxygen homeostasis is challenged in a similar way to the invasive front of tumours, and HIF-1α levels may increase in response to local hypoxia. HIF-1α in the cells invading the fat pad may induce expression of ECM-remodelling proteins known to be induced by HIF-1α and expressed in the invading mammary epithelium [1,20,21]. In addition, non-hypoxic stress may induce HIF expression in distinct phases of mammary gland development and function.

Targeted deletion of the HIF-1α gene using Cre-lox in mammary epithelial cells with the Cre recombinase expressed under the control of the MMTV promoter did not show decreased vascularisation of the mammary gland; however, differentiation and lactation were impaired [9]. Similarly, Seagroves et al. did not detect any gross differences in the pre-pregnant mammary glands of normal and HIF-1α null mice, as assessed in mammary gland whole mounts [9]. Detailed studies of how HIF-1α gene-targeted epithelial cells invade the mammary fat-pad have not been reported.

HIF-2α expression in a distinct subset of luminal epithelial cells in the milk-producing gland could be stress-induced, although local hypoxia would be expected to induce HIF-1α as well, which we did not detect (Fig 3). Other plausible forms of stress include shear stress from the milk-filled ducts and metabolic stress due to the increased need for nutrients during milk production. A lack of glucose has been shown to increase HIF-alpha levels [31,33]. Our data on HIF-2α expression does not tell us whether the HIF-2α expressing cells are a population of cells defined by their HIF-2α expression or if the expression varies over time and is dynamic; it is possible that all luminal epithelial cells experience temporal bursts of HIF-2α activity during this highly metabolically active stage. Hypoxia is primarily a metabolic stress, since the lack of oxygen limits mitochondrial ATP generation and cells must rely on the less efficient cytoplasmic glycolysis pathway to yield lactate and much less ATP per glucose molecule. Therefore, it is likely that HIF-2α expression has a specific role in the milk-producing luminal epithelium. Loss of function studies of HIF-2α in the lactating gland may help to decipher its function and establish whether HIF-2α is necessary in a subpopulation of cells or the majority of epithelial cells but for a limited time, i.e., at specific metabolic phases or in response to stress.

From a breast cancer perspective, the expression of HIF-2α during mammary gland involution is most interesting since this is a stage that involves numerous processes pivotal to carcinogenesis and cancer progression, e.g., cell death and survival, tissue remodelling, and inflammation. Infiltrating macrophages are among the HIF-2α expressing cells, but CK14-positive mammary epithelial cells also express HIF-2α. It is crucial to establish whether HIF-2α expression is helpful, and perhaps even required, for the survival of the specific population(s) of mammary epithelial cells that are needed to rebuild the mammary gland after involution so that milk production can take place after subsequent births. The process of involution, in which mammary epithelial stem and progenitor cells are primed to survive, possibly under the control of HIF-2α and during profound tissue remodelling and exposure to high hormone levels, may promote conditions that lead to the observed increased cancer risk post-partum [4]. Since we have already established that normal human breast epithelial cells acquire cancer-like traits under hypoxic conditions [7], it is tempting to speculate that the expression of the stress-induced survival factor HIF-2α during involution may contribute in such processes. To test this hypothesis HIF-2α must be selectively down-regulated in the mammary epithelial cells to spare the HIF-2α expressing macrophages that are necessary for post-lactational involution to take place [34].

**Fig 7:**
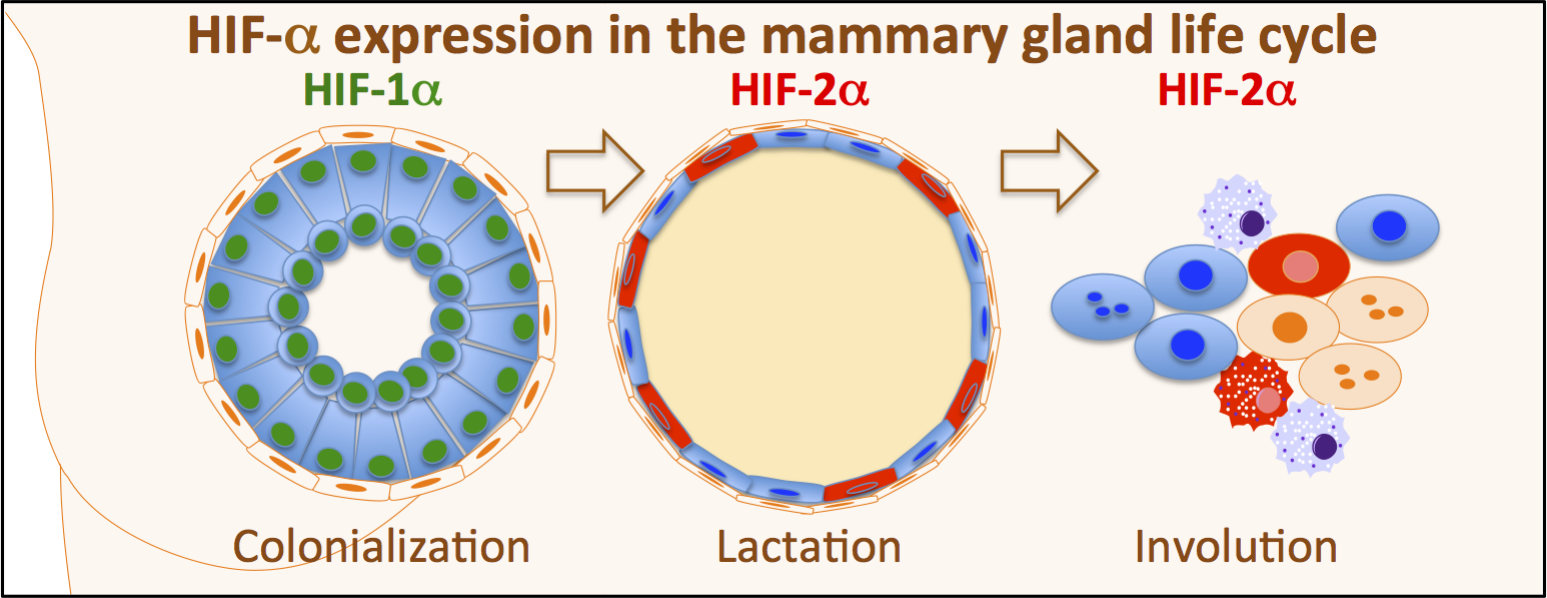
Expression of HIF-1α and HIF-2α during the life cycle of the mammary gland. HIF-1α is expressed in luminal epithelial cells of pubertal and virgin adult mice. HIF-2α shows distinct expression in dispersed lactating luminal epithelial cells. HIF-2α was expressed during post-lactational involution. During involution, CK14-positive basal or progenitor cells expressed HIF-2α

In conclusion, the expression of HIF-1α and HIF-2α takes place during specific time frames in distinct subsets of mammary epithelial cells during separate phases of mammary gland development and function (Fig 7). The divergent expression patterns of the two factors suggest that their regulation is not merely a function of oxygen availability and that HIF-1α and HIF-2α have distinct functions in mammary epithelium. These findings and the link between HIF-2α levels and breast cancer metastasis suggest that in-depth studies of HIF-1α and HIF-2α function in mammary gland development and tumourigenesis are warranted.

## References

[1] Watson CJ, Khaled WT (2008) Mammary development in the embryo and adult: a journey of morphogenesis and commitment. Development 135: 995–1003. 10.1242/dev.005439 18296651

[2] Hynes NE, Watson CJ (2010) Mammary gland growth factors: roles in normal development and in cancer. Cold Spring Harbor perspectives in biology 2: a003186 10.1101/cshperspect.a003186 20554705 PMC2908768

[3] Cardiff RD, Wellings SR (1999) The comparative pathology of human and mouse mammary glands. Journal of mammary gland biology and neoplasia 4: 105–122. 10219910 10.1023/a:1018712905244

[4] Callihan EB, Gao D, Jindal S, Lyons TR, Manthey E, Edgerton S, et al (2013) Postpartum diagnosis demonstrates a high risk for metastasis and merits an expanded definition of pregnancy-associated breast cancer. Breast cancer research and treatment 138: 549–559. 10.1007/s10549-013-2437-x 23430224 PMC3608871

[5] Bos R, Zhong H, Hanrahan CF, Mommers EC, Semenza GL, Pinedo HM, et al (2001) Levels of hypoxia-inducible factor-1 alpha during breast carcinogenesis. J Natl Cancer Inst 93: 309–314. 11181778 10.1093/jnci/93.4.309

[6] Helczynska K, Larsson AM, Holmquist Mengelbier L, Bridges E, Fredlund E, Borgquist S, et al (2008) Hypoxia-inducible factor-2alpha correlates to distant recurrence and poor outcome in invasive breast cancer. Cancer Res 68: 9212–9220. 10.1158/0008-5472.CAN-08-1135 19010893

[7] Vaapil M, Helczynska K, Villadsen R, Petersen OW, Johansson E, Beckman S, et al (2012) Hypoxic conditions induce a cancer-like phenotype in human breast epithelial cells. PloS one 7: e46543 10.1371/journal.pone.0046543 23029547 PMC3460905

[8] Helczynska K, Kronblad A, Jögi A, Nilsson E, Beckman S, Landberg G, et al (2003) Hypoxia promotes a dedifferentiated phenotype in ductal breast carcinoma in situ. Cancer Res 63: 1441–1444. 12670886

[9] Seagroves TN, Hadsell D, McManaman J, Palmer C, Liao D, McNulty W, et al (2003) HIF1alpha is a critical regulator of secretory differentiation and activation, but not vascular expansion, in the mouse mammary gland. Development 130: 1713–1724. 12620994 10.1242/dev.00403

[10] Schwab LP, Peacock DL, Majumdar D, Ingels JF, Jensen LC, Smith KD, et al (2012) Hypoxia-inducible factor 1alpha promotes primary tumor growth and tumor-initiating cell activity in breast cancer. Breast cancer research: BCR 14: R6 22225988 10.1186/bcr3087 PMC3496121

[11] Liao D, Corle C, Johnson RS (2007) Hypoxia-inducible factor-1a is a key regulator of metastasis in a transgenic model of cancer initiation and progression. Cancer Research 67: 563–572. 17234764 10.1158/0008-5472.CAN-06-2701

[12] Semenza GL (2010) Defining the role of hypoxia-inducible factor 1 in cancer biology and therapeutics. Oncogene 29: 625–634. 10.1038/onc.2009.441 19946328 PMC2969168

[13] Jiang BH, Agani F, Passaniti A, Semenza GL (1997) V-SRC induces expression of hypoxia-inducible factor 1 (HIF-1) and transcription of genes encoding vascular endothelial growth factor and enolase 1: involvement of HIF-1 in tumor progression. Cancer Res 57: 5328–5335. 9393757

[14] Zhong H, Chiles K, Feldser D, Laughner E, Hanrahan C, Georgescu MM, et al (2000) Modulation of HIF-1a expression by the epidermal growth factor/phosphatidylinositol 3-kinase/PTEN/AKT/FRAP pathway in human prostate cancer cells: implications for tumor angiogenesis and therapeutics. Cancer Res 60: 1541–1545. 10749120

[15] Seagroves TN, Peacock DL, Liao D, Schwab LP, Krueger R, Handorf CR, et al (2010) VHL Deletion Impairs Mammary Alveologenesis But Is Not Sufficient for Mammary Tumorigenesis. Am J Pathol 176: 2269–2282. 10.2353/ajpath.2010.090310 20382704 PMC2861092

[16] Rehbinder C, Baneux P, Forbes D, van Herck H, Nicklas W, Rugaya Z, et al (1996) FELASA recommendations for the health monitoring of mouse, rat, hamster, gerbil, guinea pig and rabbit experimental units. Report of the Federation of European Laboratory Animal Science Associations (FELASA) Working Group on Animal Health accepted by the FELASA Board of Management, November 1995. Laboratory animals 30: 193–208. 8843044 10.1258/002367796780684881

[17] Semenza GL, Roth PH, Fang HM, Wang GL (1994) Transcriptional regulation of genes encoding glycolytic enzymes by hypoxia-inducible factor 1. J Biol Chem 269: 23757–23763. 8089148

[18] Holmquist-Mengelbier L, Fredlund E, Löfstedt T, Noguera R, Navarro S, Nilsson H, et al (2006) Recruitment of HIF-1 alpha and HIF-2 alpha to common target genes is differentially regulated in neuroblastoma: HIF-2 alpha promotes an aggressive phenotype. Cancer Cell 10: 413–423. 17097563 10.1016/j.ccr.2006.08.026

[19] Uniacke J, Holterman CE, Lachance G, Franovic A, Jacob MD, Fabian MR, et al (2012) An oxygen-regulated switch in the protein synthesis machinery. Nature 486: 126–129. 10.1038/nature11055 22678294 PMC4974072

[20] Green KA, Lund LR (2005) ECM degrading proteases and tissue remodelingin the mammary gland. BioEssays 27: 894–903. 16108064 10.1002/bies.20281

[21] Thomasset N, Lochter A, Sympson CJ, Lund LR, Williams DR, Behrendtsen O, et al (1998) Expression of autoactivated stromelysin-1 in mammary glands of transgenic mice leads to a reactive stroma during early development. The American journal of pathology 153: 457–467. 9708806 10.1016/S0002-9440(10)65589-7 PMC1852990

[22] Semenza GL (2003) Targeting HIF-1 for cancer therapy. Nat Rev Cancer 3: 721–732. 13130303 10.1038/nrc1187

[23] Gouon-Evans V, Rothenberg ME, Pollard JW (2000) Postnatal mammary gland development requires macrophages and eosinophils. Development 127: 2269–2282. 10804170 10.1242/dev.127.11.2269

[24] Watson CJ (2006) Involution: apoptosis and tissue remodelling that convert the mammary gland from milk factory to a quiescent organ. Breast cancer research: BCR 8: 203 16677411 10.1186/bcr1401 PMC1557708

[25] Watson CJ (2006) Post-lactational mammary gland regression: molecular basis and implications for breast cancer. Expert reviews in molecular medicine 8: 1–15. 10.1017/S1462399406000196 17178008

[26] Watson CJ, Kreuzaler PA (2011) Remodeling mechanisms of the mammary gland during involution. The International journal of developmental biology 55: 757–762. 10.1387/ijdb.113414cw 22161832

[27] Lund LR, Romer J, Thomasset N, Solberg H, Pyke C, Bissell MJ, et al (1996) Two distinct phases of apoptosis in mammary gland involution: proteinase-independent and-dependent pathways. Development 122: 181–193. 8565829 10.1242/dev.122.1.181 PMC2933211

[28] Strange R, Li F, Saurer S, Burkhardt A, Friis RR (1992) Apoptotic cell death and tissue remodelling during mouse mammary gland involution. Development 115: 49–58. 1638991 10.1242/dev.115.1.49

[29] Stingl J, Eirew P, Ricketson I, Shackleton M, Vaillant F, Choi D, et al (2006) Purification and unique properties of mammary epithelial stem cells. Nature 439: 993–997. 16395311 10.1038/nature04496

[30] Jögi A, Øra I, Nilsson H, Lindeheim A, Makino Y, Poellinger L, et al (2002) Hypoxia alters gene expression in human neuroblastoma cells toward an immature and neural crest-like phenotype. Proc Natl Acad Sci U S A 99: 7021–7026. 12011461 10.1073/pnas.102660199 PMC124521

[31] Nilsson H, Jögi A, Beckman S, Harris AL, Poellinger L, Påhlman S (2005) HIF-2alpha expression in human fetal paraganglia and neuroblastoma: relation to sympathetic differentiation, glucose deficiency, and hypoxia. Exp Cell Res 303: 447–456. 15652356 10.1016/j.yexcr.2004.10.003

[32] Tian H, Hammer RE, Matsumoto AM, Russell DW, McKnight SL (1998) The hypoxia-responsive transcription factor EPAS1 is essential for catecholamine homeostasis and protection against heart failure during embryonic development. Genes Dev 12: 3320–3324. 9808618 10.1101/gad.12.21.3320 PMC317225

[33] Williams KJ, Telfer BA, Airley RE, Peters HP, Sheridan MR, van der Kogel AJ, et al (2002) A protective role for HIF-1 in response to redox manipulation and glucose deprivation: implications for tumorigenesis. Oncogene 21: 282–290. 11803471 10.1038/sj.onc.1205047

[34] O'Brien J, Martinson H, Durand-Rougely C, Schedin P (2012) Macrophages are crucial for epithelial cell death and adipocyte repopulation during mammary gland involution. Development 139: 269–275. 10.1242/dev.071696 22129827 PMC13621425

